# Endothelial Differentiation of Mesenchymal Stromal Cells

**DOI:** 10.1371/journal.pone.0046842

**Published:** 2012-10-04

**Authors:** Karolina Janeczek Portalska, Anne Leferink, Nathalie Groen, Hugo Fernandes, Lorenzo Moroni, Clemens van Blitterswijk, Jan de Boer

**Affiliations:** MIRA Institute for Biomedical Technology and Technical Medicine, University of Twente, Enschede, Overijssel, The Netherlands; Instituto Butantan, Brazil

## Abstract

Human mesenchymal stromal cells (hMSCs) are increasingly used in regenerative medicine for restoring worn-out or damaged tissue. Newly engineered tissues need to be properly vascularized and current candidates for *in vitro* tissue pre-vascularization are endothelial cells and endothelial progenitor cells. However, their use in therapy is hampered by their limited expansion capacity and lack of autologous sources. Our approach to engineering large grafts is to use hMSCs both as a source of cells for regeneration of targeted tissue and at the same time as the source of endothelial cells. Here we investigate how different stimuli influence endothelial differentiation of hMSCs. Although growth supplements together with shear force were not sufficient to differentiate hMSCs with respect to expression of endothelial markers such as CD31 and KDR, these conditions did prime the cells to differentiate into cells with an endothelial gene expression profile and morphology when seeded on Matrigel. In addition, we show that endothelial-like hMSCs are able to create a capillary network in 3D culture both *in vitro* and *in vivo* conditions. The expansion phase in the presence of growth supplements was crucial for the stability of the capillaries formed *in vitro*. To conclude, we established a robust protocol for endothelial differentiation of hMSCs, including an immortalized MSC line (iMSCs) which allows for reproducible *in vitro* analysis in further studies.

## Introduction

Human mesenchymal stromal cells (hMSCs), also referred to as colony forming unit-fibroblasts (CFU-F), mesenchymal stem cells, or mesenchymal progenitor cells were first identified as a subpopulation of bone marrow cells by Friedenstein [1]. Their abundance among other bone marrow cells was estimated to be 0.01% to 0.001% [1]–[3]. Later research showed that MSCs can be isolated from other types of tissues as well, including adipose tissue, placenta, periosteum, trabecular bone and femur [4]–[7]. MSCs can be characterized based on their fibroblast-like morphology and ability to differentiate into various cell types [8]. To induce adipogenic differentiation, stimulation with insulin, dexamethasone and IBMX is typically applied [9]. MSCs cultured in dexamethasone differentiate towards osteoblasts and can participate in new bone formation after implantation in critical size bone defects [10]–[12]. TGF-β stimulation, especially when combined with BMP-2 treatment, can trigger chondrogenic differentiation of MSCs [13]. While MSC differentiation towards adipo-, osteo- and chondrogenic lineages is widely investigated, some studies have also shown that MSCs can differentiate towards muscular and neural phenotype, but those are less documented and the differentiation protocols are not yet widely applied. For instance, myoblasts can be obtained from MSCs after applying basic fibroblast growth factor (bFGF) and forskolin [14]–[16]. On the other hand, platelet-derived growth factor (PDGF) together with forskolin and glial growth factor (GGF-2) stimulation results in differentiation of MSCs into cells with a Schwann cell-like phenotype [17]. Continuous trials to obtain neural cells have resulted in several studies demonstrating the possibility of obtaining MSC-derived cells that can support the regeneration of nerves and participate among others in therapy of erectile dysfunction, multiple sclerosis and spinal cord injury [18], [19]. MSCs are also capable of suppressing allo-responses and appear to be non-immunogenic [20]. Therefore, hMSCs are increasingly used in regenerative medicine as a source of cells for restoring worn-out or damaged tissues such as cartilage, cardiac muscle or bone. In the field of bone tissue engineering there are a total of 9 human clinical trials performed to date [21]. Other clinical trials with MSCs are performed to improve cardiac functions after myocardial infarction [22], [23] and to restore liver and kidney function after failure [24], [25].

The standard approach in regenerative medicine when MSCs are used is to identify the cell type necessary for the therapy and then differentiate MSCs towards this phenotype. Differentiated cells are then used in animal models and when the therapy is successful, clinical trials are performed. There is, however, a clear need for endothelial cells (EC) in this approach. ECs are needed to line artificial vessels and to restore vascularization of ischemic tissues. This is a crucial point in therapy of peripheral vascular diseases [26] which is a growing medical problem in Western societies and manifests itself in obstruction of large arteries. This leads to retraction of small arteries and capillaries followed by acute or chronic ischemia in surrounding tissues. Only the recovery of the vascular network in such tissues can restore blood flow and prevent limb amputation [27]. Another difficulty that may be solved by providing a good source of endothelial cells is the maintenance of cell survival after graft implantation. The key parameter in this problem is the supply of nutrients and oxygen in which diffusion is a limiting factor. Metabolically active cells must be situated within 150–200 µm distance from blood vessels in order to maintain proper functions [28], [29]. To maintain cell survival in large grafts, a vascular structure needs to be introduced within the graft which can rapidly connect to the host’s blood vessels upon implantation. Without that, cell death and lack of new tissue formation in the interior of the implant will occur [30]. The proof of principle for the application of ECs in the abovementioned conditions has been achieved using endothelial progenitor cells (EPCs) [31] and endothelial cells. EPCs are immature cells capable of differentiating into mature endothelial cells *in vitro* and *in vivo* [32]. EPCs are cells with some self-renewing capacity present both in the bone marrow and in circulation [33]. Recently, the vasculogenic potential of different EPCs was investigated and compared [34], [35]. EPCs adhere to gelatin and fibronectin, take up acetylated LDL, bind lectins from *Ulex Europaeus*, and express marker proteins of the EC lineage (e.g. CD31, KDR, vWF). However, they either lose their potential after prolongated expansion or their expansion capacity is not enough to provide sufficient numbers of cells for therapeutic applications [36], [37]. Mature ECs isolated from umbilical vein or aorta are considered as another cell source for graft vascularization [38]. These cells can be expanded in vitro and perform well in creating vascular networks *in vivo* [39], [40]. Autologous isolation is however only possible by sacrificing a current vessel of the patient. Since bone marrow derived MSCs were shown to differentiate into adipogenic, osteogenic and chondrogenic lineages [41] they can also be considered as a promising source for obtaining endothelial cells that are able to create vascular networks. There are several factors reported to influence endothelial differentiation and maintain endothelial potential *in vitro* while using embryonic stem cells or EPCs. Specifically, the effects of endothelial growth supplements [42]–[44], shear forces [45]–[49] and composition of extracellular matrix [44], [50] are important factors in EC culture. These factors influence the efficiency of capillary-like structure formation on Matrigel, endothelial markers expression (CD31, vWF, KDR), the ability to take up acetylated LDL and their *in vivo* performance. In the last 10 years, much effort was put into establishing protocols for endothelial differentiation of mesenchymal stromal cells. In particular, cells isolated form adipose tissue were reported to respond positively for endothelial differentiation [51], [52]. In the case of MSCs isolated form bone marrow (BM-MSCs) several studies were conducted with various outcomes. The work of Oswald *et al.* shows that BM-MSCs can acquire *in vitro* phenotypic and functional features of ECs [53]. Silva *et al.* demonstrated that MSCs injected into ischemic myocardium can differentiate into smooth muscle cells and endothelial cells *in vivo*, resulting in increased vascularity and improved cardiac functions [54]. Differentiation of MSCs into endothelial cells *in vitro* did not improve their performance *in vivo* [55], which could be due to a sub-optimal differentiation protocol, similar to previous attempts to improve ectopic bone formation by hMSCs. Only after precise adjustment of several parameters a protocol was obtained that resulted in an improvement *in vivo* [56]. In this manuscript we demonstrate a robust and efficient endothelial differentiation protocol for MSCs, which describes the isolation, expansion and differentiation of BM-MSCs and their potential in tissue engineering as endothelial-like cells.

## Materials and Methods

### Isolation and Culture

Human mesenchymal stromal cells (hMSCs) were isolated from human bone marrow from donors with written informed consent [57]. Aspirates were resuspended using a 20G needle and plated at a density of 0.5 million mono-nucleated cells per cm^2^. Cells were grown in MSC proliferation medium which contains minimal essential medium (alfa-MEM, GIBCO) supplemented with 10% fetal bovine serum (FBS, Lonza), 100 U/ml penicillin (GIBCO), 10 µg/ml streptomycin (GIBCO), 2 mM L-glutamin (GIBCO), 0.2 mM L-ascorbic acid 2-phosphate magnesium salt (ASAp, Sigma-Aldrich) and 1 ng/ml bFGF (Fisher Scientific) at 37°C in a humid atmosphere with 5% CO2. Cells were expanded up to passage 2. For further experiments hMSCs from two different donors and one immortalized clone (iMSCs, courtesy of Ola Myklebost, University of Oslo, Norway) were cultured in basic medium (alfa-MEM supplemented with 10% FBS, 100 U/ml penicillin, 10 µg/ml streptomycin, 2 mM L-glutamin and 0.2 mM ASAp). Human umbilical vein endothelial cells (HUVEC, Lonza) were cultured in endothelial growth medium (EGM-2, Lonza). Mouse skeletal myoblast cells (C2C12) and mouse embryonic fibroblasts (MEF) (Cell Essentials) were cultured in Dulbecco’s Modified Eagle’s Medium (DMEM, GIBCO) supplemented with 10% FBS, 100 U/ml penicillin and 10 µg/ml streptomycin.

### Endothelial Induction of MSCs

hMSCs from passage 2 and iMSCs from passage 25 were used for the endothelial induction protocol. Cells were seeded at a density of 3,000 cells/cm^2^ on tissue culture plastic in EGM-2 and cultured for 10 days. After one day in static culture shear force was applied using an XYZ shaker (3D shaker). Cultures were rotated at a rate of 20 rpm. Cells that were cultured according to this protocol will be referred to as pre-differentiated MSCs.

Phalloidin and DAPI stainings were used for cell size and shape analysis. Pictures were taken with a BD Pathway™ Bioimager and analyzed using Attovision software. A minimal number of 300 cells per condition were analyzed.

For induction on Matrigel, wells of 6-well plates were covered with 1 ml of growth factor reduced Matrigel (BD Bioscience) diluted 1∶1 in EGM-2 without growth factors. Cells were seeded at a density of 30,000 cells/cm^2^ and cultured in a humid atmosphere with 5% CO2 for 24 hours. The formation of capillary-like structures (CLS) was observed over time using an inverted microscope (Nikon Eclipse TE300). Pictures were taken at different time points using a Nikon DS-L2 camera.

### Wound Healing Assay

iMSCs were cultured for 10 days in EGM-2 on an XYZ shaker and were then trypsinized and seeded in 6-well plates at a density of 15,000 cells/cm^2^. iMSCs expanded in basic medium were used as a negative control. After 24 h of culture, cells reached 90% confluency. The cell monolayers were gently scratched using a pipette tip across the entire diameter of the well. Cells were washed twice with PBS to remove cellular debris and then cultured in basic medium to avoid differential growth factor stimulation during the assay. The size of the wounds directly after making the scratch and following wound closure was observed over time using an inverted microscope (Nikon Eclipse TE300). Pictures were taken at each time-point using a NikonDS-L2 camera. The wound size was determined using TScratch software (CSElab) as percentage of the picture area that was not occupied by cells.

### TubeCount

**Figure 1:**
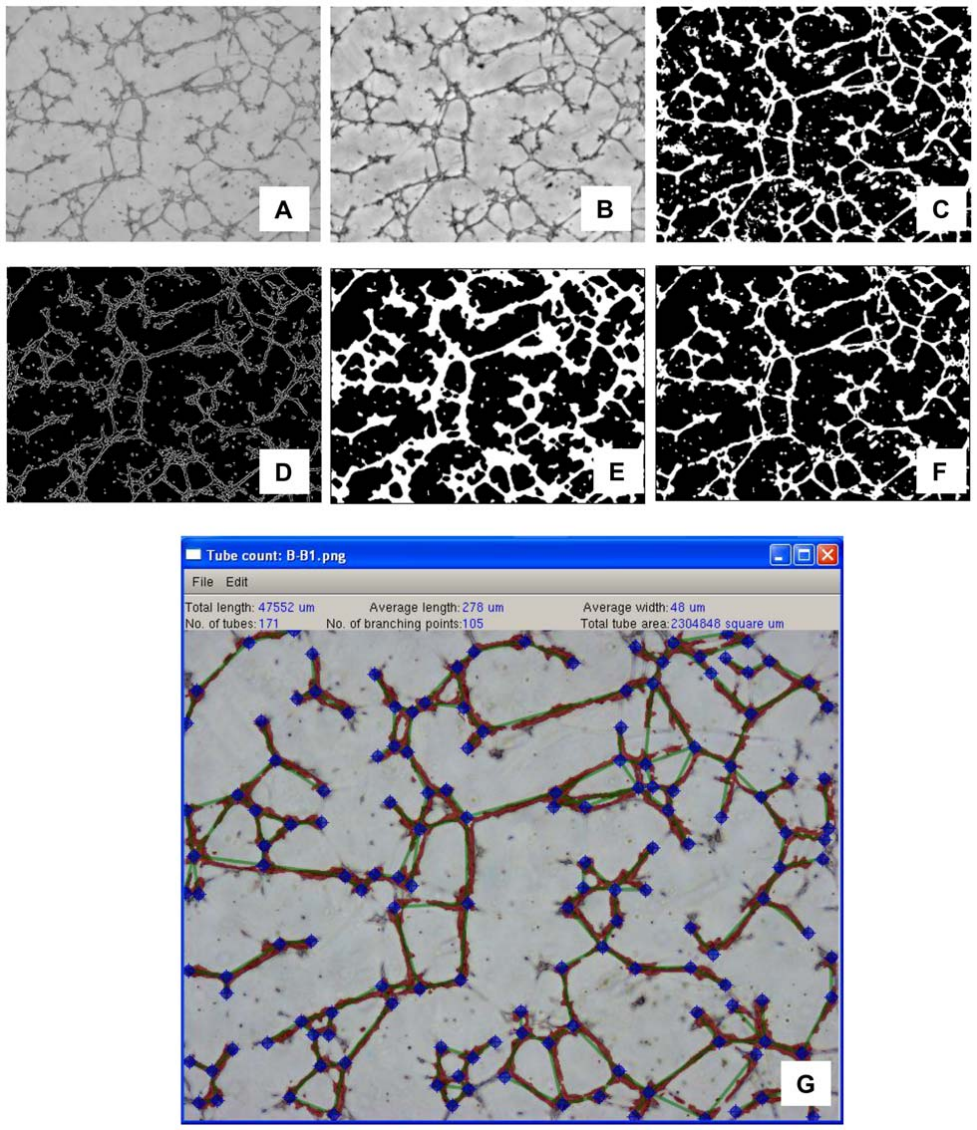
Predifferentiation of iMSCs. Cell shape after 10 days of culture in basic medium (A) and in EGM-2 (B). Average cell area (C) was compared. Cell shape was characterized based on circularity coefficient (D). Error bars represents 95% confidence interval, * denotes statistical significance (P<0.05).

Custom image recognition and analysis software has been implemented in C++ programming language with the aid of dlib library (http://dlib.net/) used for image processing and graphical user interface, Anti-Grain Geometry library (http://www.antigrain.com/) as vector graphics engine and FFTW library (http://www.fftw.org/) for calculating Fourier transforms. The purpose of the software is to quantify tube formation efficiency which is performed in two stages. The first stage is a fully automated segmentation routine which partitions the image into background region (insignificant to further analysis) and foreground region (containing cells). Segmentation is based on the assumed characteristics of cell images obtained with phase contrast microscopy (Fig. 1A) and primarily relies on the existence of well-defined cell edges and on low pixel intensity (dark shades) within the cells as compared to image background. To extract regions which fulfill these criteria the image is first enhanced using generic image quality improvement methods such as homomorphic filtering, median-based noise removal and contrast stretching (Fig. 1B). Such enhanced images are then treated with a localized intensity thresholding algorithm extracting pixels which have lower-than-average intensity with the average intensity calculated over a window of a specified size (Fig. 1C). The algorithm is repeated for multiple window sizes and the final result is an intersection of the results obtained in all iterations. Thus, the pixels which show below-average intensity regardless of averaging window size are classified as potential foreground pixels. On the other hand, the enhanced cell image is also processed using a Sobel edge detector in order to find cell boundaries (Fig. 1D). The detected edges are processed using a fill/connect algorithm which connects neighboring edges resulting in a uniform binary bitmap containing pixels corresponding to objects with distinct edges (Fig. 1E). An intersection of this binary bitmap with the one obtained with intensity thresholding is assumed to be a correct foreground mask (Fig. 1F).

The second stage consists of manual tube identification where the software user depicts beginning and ending points of individual tubes on the processed image. As result of this process a tube topology graph is formed which, in combination with the information obtained in the segmentation stage, enables to gather valuable statistics such as total and average tube length, average tube width, number of tube branching points and total tube area (Figure 1G).

### EL-MSC Characterization

#### RNA isolation and quantitative PCR

Total RNA was isolated using TRIZOL reagent according to the manufacturer’s protocol. Briefly, 1 ml of Trizol reagent was added per T25 flask (cells cultured in basic medium) or per well (cells cultured on Matrigel in 6-well plates). Samples were incubated for 5 min at room temperature to allow complete dissociation. Phase separation was performed by adding chloroform, and then samples were shaken vigorously for 15 seconds and incubated for 3 min at room temperature. After that samples were centrifuged at 12,000×g for 15 min. RNA was precipitated by mixing the aqueous phase with isopropyl alcohol followed by 10 min incubation at room temperature. Samples were centrifuged again and the remaining RNA pellet was washed with 75% ethanol. The obtained samples were dissolved in water. The quantity and quality of RNA was analyzed using spectrophotometry (ND-1000 spectrophotometer.

For first strand cDNA synthesis 500 ng of RNA was used in combination with Superscript II (Invitrogen) according to the manufacturer’s protocol. One ul of 3× diluted cDNA was used for further gene amplification. PCR was performed in a Light Cycler real time PCR machine (BioRad). Data was analyzed using Bio-Rad iQ5 software. Expression of endothelial genes was calculated relative to GAPDH levels by the comparative ΔCT method. Primers used in the study are listed in Table 1.

**Table 1:** Primers used for qPCR.

CD31 (Platelet Endothelial Cell Adhesion Molecule-1)	F 5′ TCTATGACCTCGCCCTCCACAAA 3′
	R 5′ GAACGGTGTCTTCAGGTTGGTATTTCA 3′
KDR (VEGF receptor 2)	F 5′ ACTTTGGAAGACAGAACCAAATTATCTC 3′
	R 5′ TGGGCACCATTCCACCA 3′
vWF (von Willebrand factor)	F 5′ TGCTGACACCAGAAAAGTGC 3′
	R 5′ AGTCCCCAATGGACTCACAG 3′
GAPDH	F 5′ CGCTCTCTGCTCCTCCTGTT 3′
	R 5′ CCATGGTGTCTGAGCGATGT 3′

### Immunostaining

Cells for immunostaining were fixed with 70% ethanol and permeabilized with 0.01%Triton-X. To block non-specific background staining, cells were incubated with 5% BSA (Sigma- Aldrich) in PBS for 30 min. Cells were then incubated with mouse anti-human CD31 antibody (Dako) or with rabbit anti-human VEGFR2 (Cell Sygnaling) for 2 hours. Cells were washed in PBS with 1% BSA and subsequently incubated with the secondary antibody (AlexaFluor 488 conjugated goat anti mouse or AlexaFluor 594 conjugated goat anti rabbit, Invitrogen) for 1 hour. Cells were washed and counterstained with DAPI and photographed with a BD Pathway™ Bioimager.

#### Acetylated low-density lipoprotein (ac-LDL) uptake assay

iMSCs were cultured in EGM-2 on an XYZ shaker for 10 days, then transferred to Matrigel and cultured for another 24 hours. Cells were then recovered from Matrigel by 30 min incubation in a 1∶1 mixture of 0.25% trypsin and dispase (BD Bioscience). Recovered cells were seeded in 96-well plates and cultured in EGM-2 for one more day. Subsequently, cells were incubated for 4 hours in EGM-2 supplemented with 10 ug/ml Dil-labeled ac-LDL (Invitrogen) at 37°C in a humid atmosphere. Finally, cells were washed with PBS 3 times.

**Figure 2:**
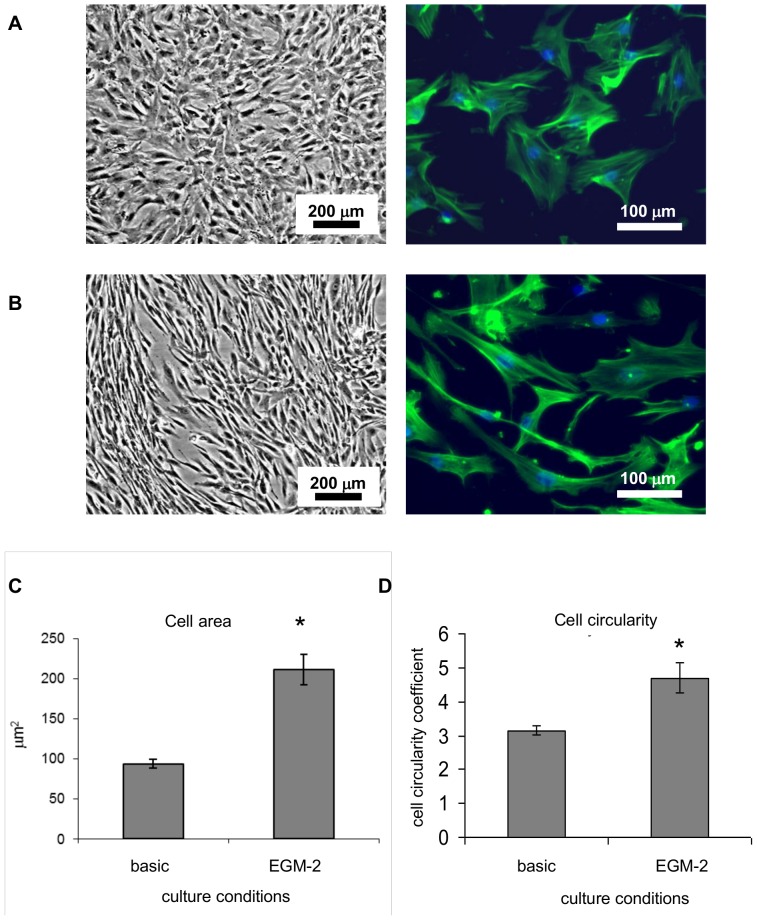
Wound healing assay. Pictures were taken directly after making the wound, 12 and 24 hours later (A). Quantification of wound recovery was performed in TScratch program and presented on the graph (B). Error bars represent standard deviation, * denotes statistical significance (P<0.05).

### Biodegradable Scaffold Preparation

Porous scaffolds composed of a 50/50 blend of poly-(l-lactic acid) (PLLA) and polylactic-glycolic acid (PLGA) were fabricated by a salt-leaching process. PLLA (Polysciences) and PLGA (Sigma-Aldrich) were dissolved 1∶1 in chloroform (Fisher Scientific) with a final concentration of 5% (wt/vol). Two ml of this solution was poured into Teflon containers (Savillex) with a diameter of 50 mm and homogenized with 3.4 g of sodium chloride particles with an average grain size of 425 µm. The solvent was evaporated overnight under nitrogen flow. To leach the salt, the obtained polymer films were immersed in distilled water for 6 hours (changed every hour). The leached films with a thickness of 500 µm and a pore size of 200±60 µm were lyophilized overnight and subsequently cut in circular disks with a diameter of 5 mm. Before culture the scaffolds were sterilized in 70% ethanol for 2 days, washed three times with PBS and incubated in culture medium overnight.

**Figure 3:**
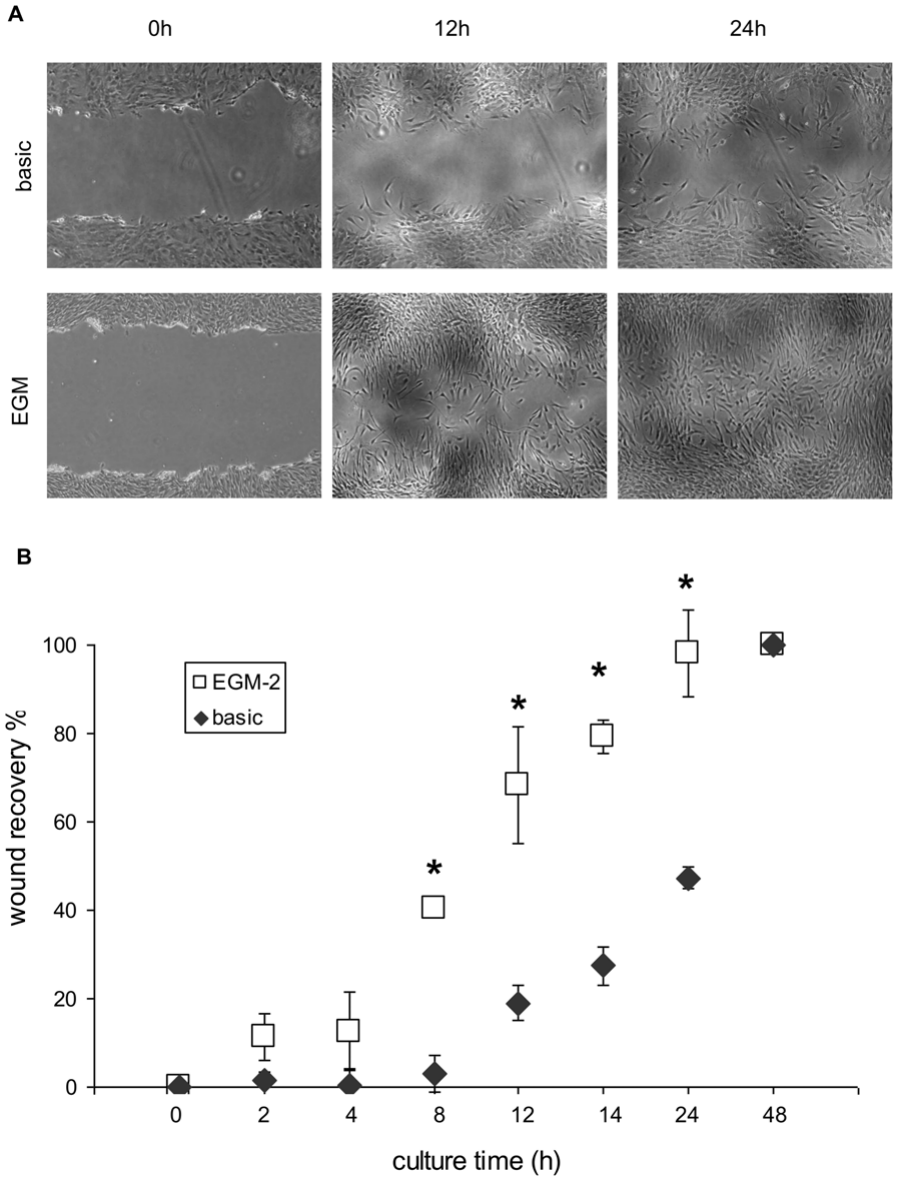
Capillary-like structures formation on Matrigel. EL-hMSCs, EL-iMSCs and HUVECs were cultured on Matrigel for 24 h in EGM-2 medium (A). Total tube length, total tube area and number of branching points were compared (B). * and ** denotes statistical significance (P<0.05).

### PLLA/PLGL Construct Preparation and Implantation

Constructs for implantation were prepared as described before [58]. Briefly, 500,000 cells were pooled and resuspended in 20 µl of a 1∶1 mixture of EGM-2 medium without growth factors and growth factor reduced Matrigel. This suspension was applied onto the scaffold and allowed to be absorbed and solidify for 30 min at 37°C in a humid atmosphere with 5% CO_2_. Culture medium (EGM-2) was then added; scaffolds were detached from the wells and cultured further on a shaker at 37°C in a humid atmosphere with 5% CO2. Medium was changed every other day. After 10 days of culture samples were fixed in 10% formalin or implanted into mice.

**Figure 4:**
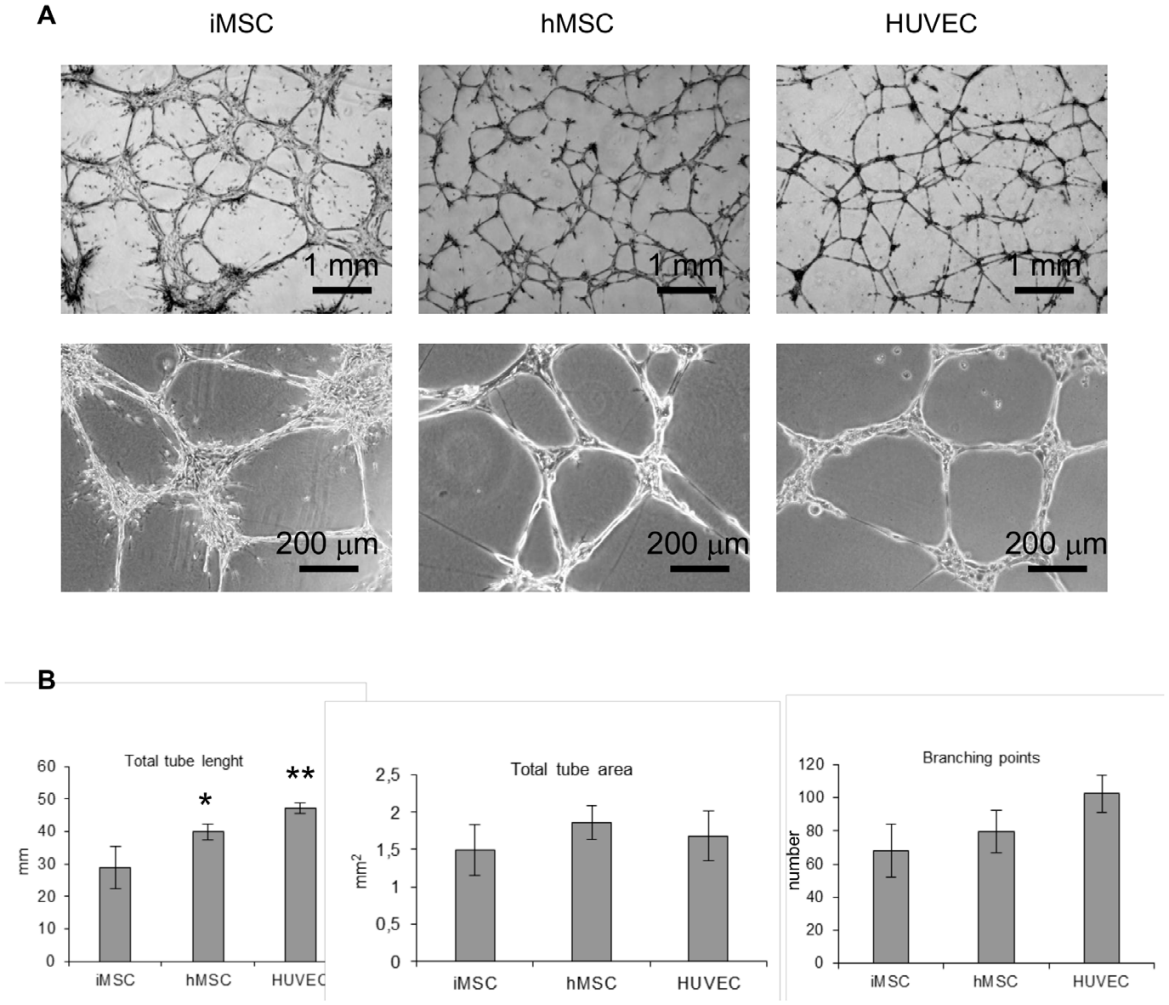
Capillary-like structures formation on Matrigel. Time course study. iMSCs were cultured for 10 days in basic or EGM-2 medium and then seeded on Matrigel in basic or EGM-2 medium. Dynamics of capillaries formation was observed for following 7 days.

Male 6-week old NMRI-nu mice (Harlan) were anesthetized with a mixture of isofluorane and oxygen after which constructs were subcutaneously implanted in four pockets. Animals were housed at the Central Laboratory Animal facility (Utrecht University, Utrecht, The Netherlands), and experiments were approved by the local animal care and use committee. Two weeks after implantation mice were sacrificed and implants were recovered. Samples were fixed in 10% formalin, embedded in paraffin and sectioned at 5 µm before staining.

### Histochemical Staining and Image Analysis

Hematoxylin (Sigma-Aldrich) and eosin (Sigma-Aldrich) staining as well as Masson’s trichrome (Merck Chemicals) staining were performed according to manufacturers’ protocols. Samples were photographed using a Nikon Elipse E600 microscope. Based on Masson’s trichrome stainings vessels were counted manually by 4 observers blinded to the sample composition. Three areas of each sample (10 samples per condition) were used for this quantification.

**Figure 5:**
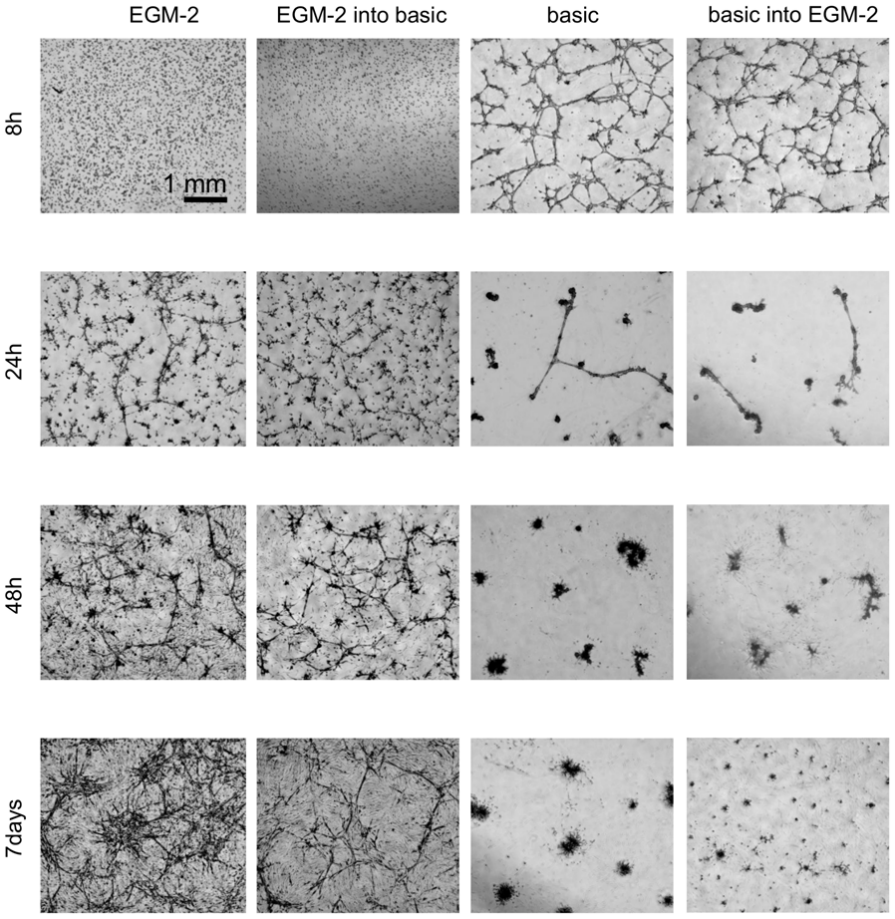
Image segmentation steps. Original image (A), quality enhancement (B), intensity thresholding (C), edge detection (D), edge connection/filling (E), final segmentation result (F). Print Screen of the analysis of tube topology (G).

For detecting endothelium of human origin CD31 staining was performed. Antigen retrieval was achieved with IHC-Tek Epitope Retrieval Solution (IHC World). Sections were then incubated with mouse-anti-human CD31 primary antibody (Dako), which does not cross-react with mouse tissue. Following this, biotinylated horse anti-mouse secondary antibody (Antibodies-online.com) was applied. Slides were developed with Labeled Streptavidin Biotin (LSAB) with DAB Chromogen (IHC World) and weakly counterstained with Mayer’s hematoxylin (Sigma-Aldrich).

### Statistics

Each experiment was performed in triplicate. The data was analyzed using Student’s t-test at p<0.05. Data that required multiple comparison test was analyzed in SPSS (PASW statistics) using one-way Anova followed by Tukey’s multiple comparison test (P<0.05). Error bars on graphs represent standard deviation or 95% confidence interval as indicated in the graph legends.

**Figure 6:**
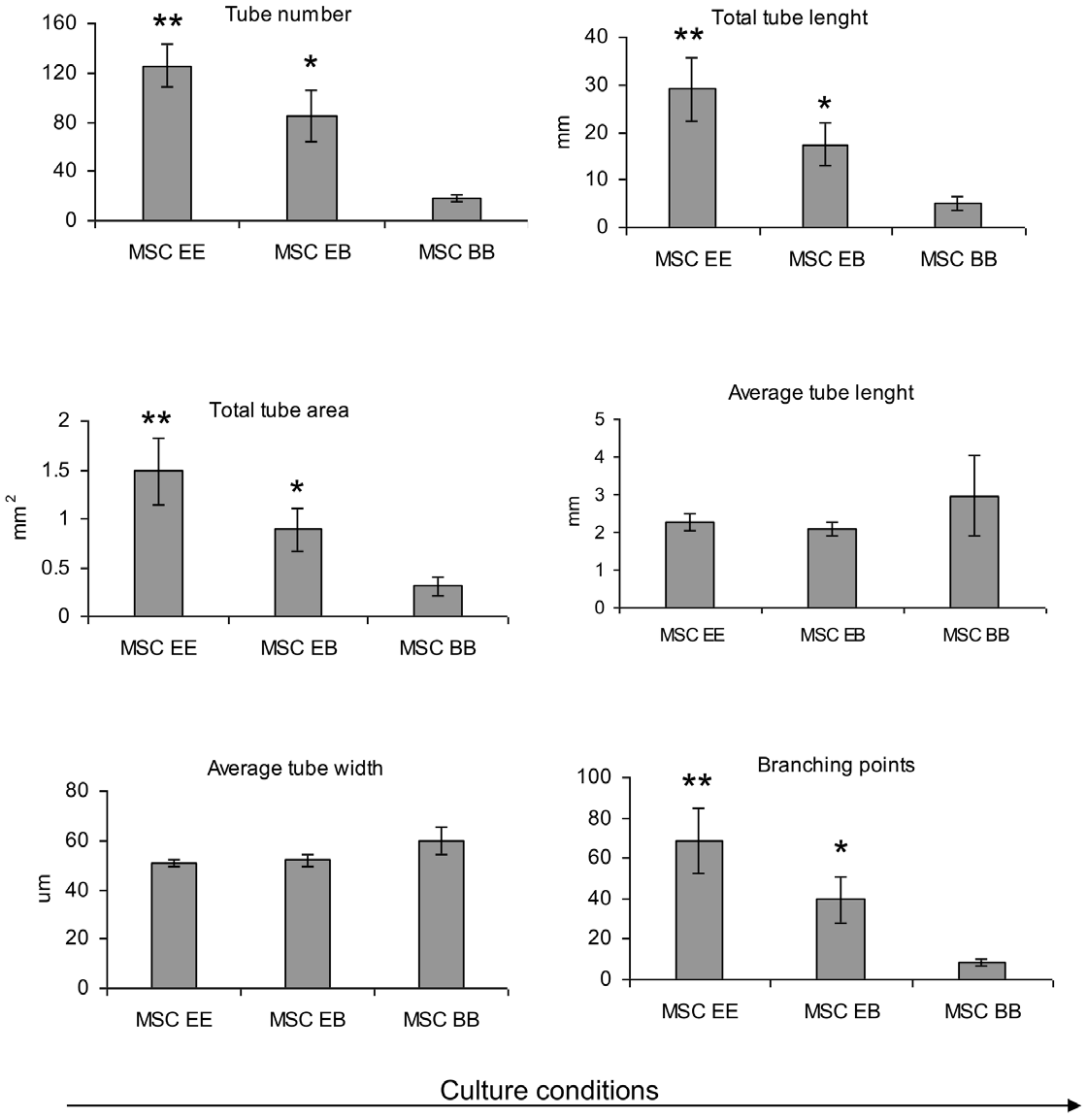
Quantification of capillary-like structures on Matrigel. Quantification of tube formation by cells cultured in 3 different conditions: cells predifferentiated in EGM-2 and seeded on Matrigel in the same medium (EE), cells predifferentiated in EGM-2 and seeded on Matrigel in basic medium (EB) and naïve MSCs expanded in basic medium and seeded on Matrigel in basic medium (BB). Graphs show total tube length, average tube length and average tube width, total tube area, number of tubes and number of branching points per picture. Error bars represent standard deviation, * and ** denotes statistical significance (P<0.05) towards all other bars.

### Ethics Statement

Human mesenchymal stromal cells (hMSCs) were isolated from human bone marrow from donors with written informed consent. This study was carried out in strict accordance with the recommendations of Medisch Ethische ToetsingsCommissie Twente (Medical Ethical Research Committee Twente) and was approved by this Committee.

The animal study reported on in this manuscript was ethically assessed a priori by an animal ethics committee 2010-III-10-125 DEC-Utrecht. Animals were housed at the Central Laboratory Animal facility (Utrecht University, Utrecht, The Netherlands), and experiments were approved by the local animal care and use committee Dierexperimentencommissie Academisch Biomedisch Centrum (DEC-ABC). All surgery was performed under isofluorane/oxygen anesthesia, and all efforts were made to minimize suffering.

**Figure 7:**
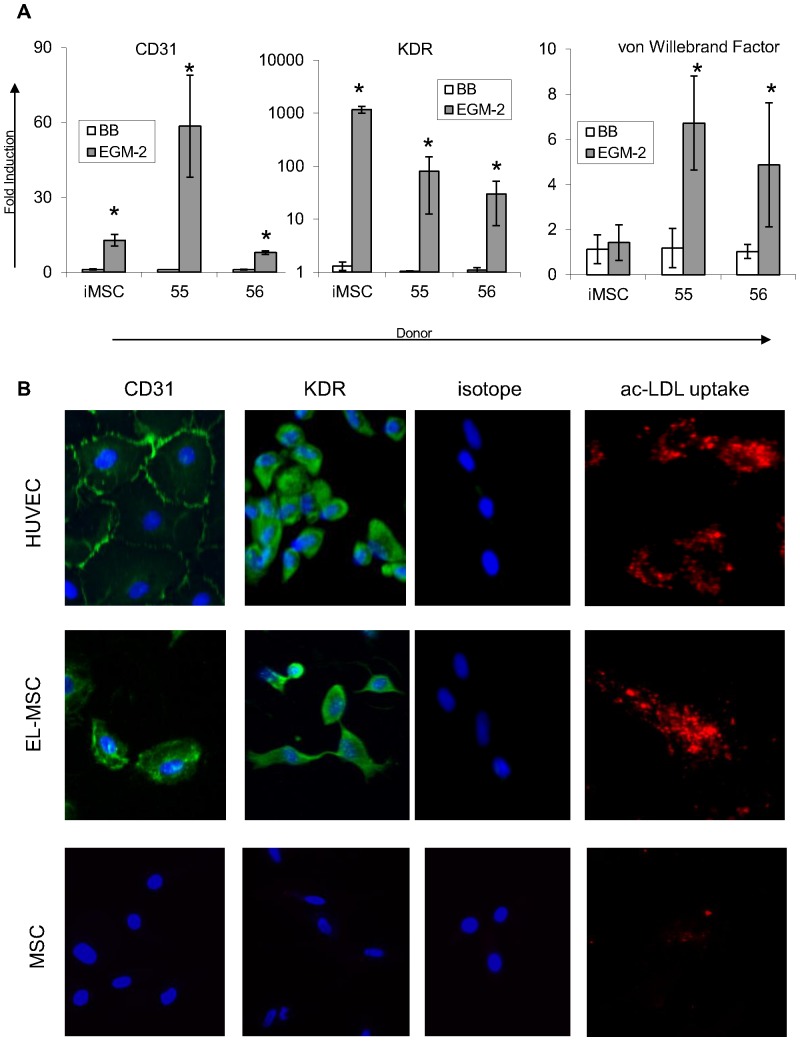
Endothelial marker expression in differentiated MSCs. Gene expression profiles of endothelial markers in EGM-2 and Matrigel culture (EE) (A). Expression is indicated as fold induction compared to cells grown in basic medium (BB) on plastic and normalized to GAPDH RNA. hMSCs from donors 55 and 56 and iMSCs were used in this experiment. Error bars represent standard deviation, * denotes statistical significance (P<0.05). Staining for endothelial markers (B).

## Results

### Endothelial Differentiation Medium Effects Cell Shape

Because EGM-2 induces endothelial differentiation in human amniotic fluid-derived stem (AFS) cells, [46] we decided to use this medium to differentiate MSCs towards an endothelial-like phenotype. First, we analyzed whether MSCs cultured in EGM-2 acquired an endothelial-like phenotype. HUVEC were used as positive control for expression of endothelial markers. Cytoskeleton staining and qPCR study showed that the observed change of shape and size of pre-differentiated MSCs was not followed by expression of endothelial specific markers such as CD31, KDR or vWF (data not shown). Additionally, MSCs grown in abovementioned conditions did not take up ac-LDL (data not shown). We did observe a difference in cell shape between MSCs cultured in EGM-2 and basic medium. Cells cultured in EGM-2 were clearly more elongated than MSCs cultured in basic medium but exact measurements of cell shape and area were difficult due to the high cell density. To quantify this phenomenon, sub-confluent cell culture was necessary. Cells were trypsinised and seeded at lower density and further growth was allowed for 2 more days to ensure cell spreading. Morphology of naïve MSCs showed a typical fibroblast-like shape in contrast to cells grown in EGM-2 (Fig. 2A–B). The average size of MSCs cultured in basic medium was approximately 100 µm^2^ and the average cell circularity was 3 (circularity of 1 represents round objects, the higher the coefficient is, the less round object it describes). The shape of pre-differentiated MSCs was significantly altered: the area increased to approximately 200 µm^2^ and the circularity coefficient was close to 5, suggesting that cells grown in EGM2 were more elongated than naïve MSCs (Fig. 2C–D). The observed phenomenon was opposite to what was expected because MSCs from other sources [59] acquired a cobblestone-like morphology typical for endothelial cells.

**Figure 8:**
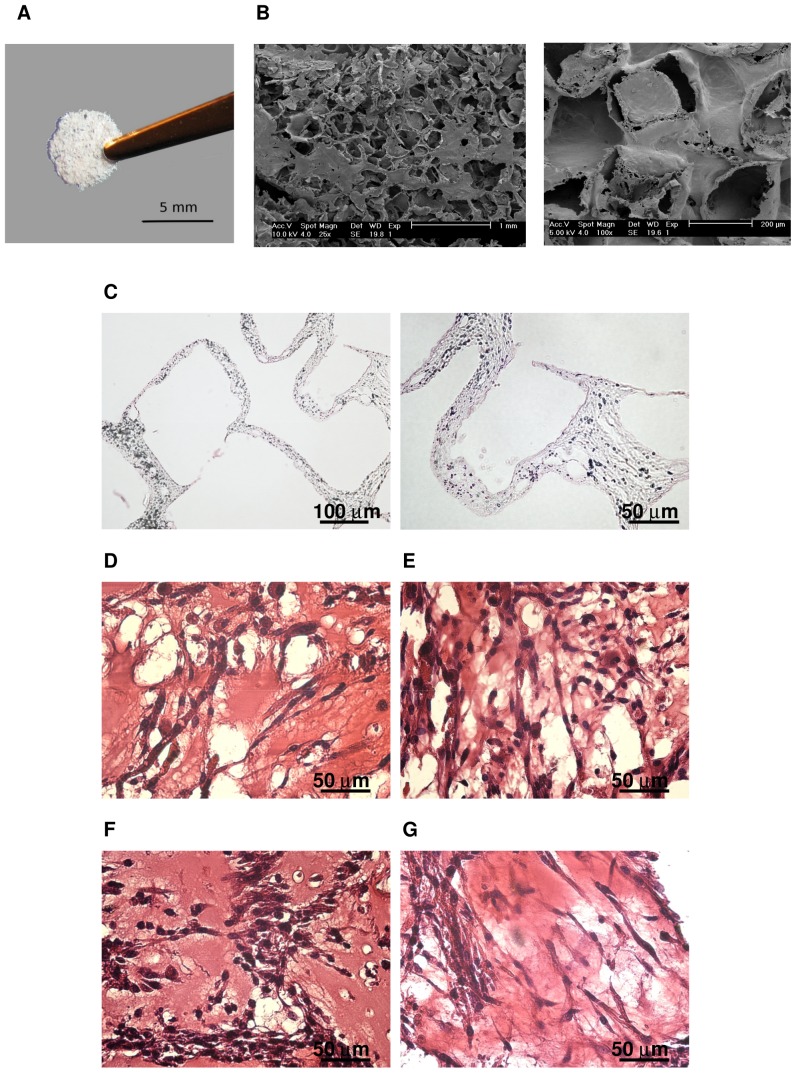
In vitro vascularization of polymeric construct combined with Matrigel and cells. PLLA/PLGL scaffold before cell seeding (A). Scanning electron microscope (SEM) pictures of scaffold taken after gold coating (B). Eosin/hematoxylin staining of tissue sections taken from constructs after 10 days of in vitro culture without cells (C), with C2C12 (D), with C2C12 and MSCs (E), with C2C12 and HUVECs (F), with C2C12 and EL-MSCs (G).

### Wound Healing Assay

The wound-healing assay is a simple method to study directional cell migration in vitro [60]. Migration of vascular endothelial cells plays an important role in vasculogenesis and angiogenesis [61]. We used the scratch wound healing assay of tissue-culture cell monolayers to assess the migration potential of predifferentiated MSCs. The wound recovery increased over time and the rate of this was taken as a measure of cell migration (Fig. 3A). We observed that 10 days culture in EGM-2 significantly increased the migration rate of MSCs (Fig. 3B). The size of the wound in EGM-2-cultured MSC monolayer was reduced by 40% after 8 hours and the wound closed completely after 24 hours. Naïve MSCs needed 24 hours for a 45% reduction in wound size and 48 hour to close the wound completely. This assay showed that MSCs cultured in EGM-2 migrate faster than naïve MSCs.

### Capillary-like Structures Formation

The angiogenic capability of various cell types was assessed using an *in vitro* capillary formation assay on Matrigel. Three cell types were used for this study: HUVEC which served as positive control, bone-marrow derived hMSCs and iMSCs. We decided to test whether iMSCs react in a similar way to hMSCs to serve as a cell source for future studies without problems associated with donor variation. As shown in Fig. 4A, cells from each cell type performed with similar efficiency. Measurement of total tube length (Fig. 4B) revealed significant but not critical differences between iMSCs and hMSCs and HUVECs. Total tube area was similar in all three cases and the number of branching points did not reveal significant differences between iMSCs, hMSCs and HUVECs. Comparison of all those parameters allowed us to use iMSCs as a model of MSCs for further study.

**Figure 9:**
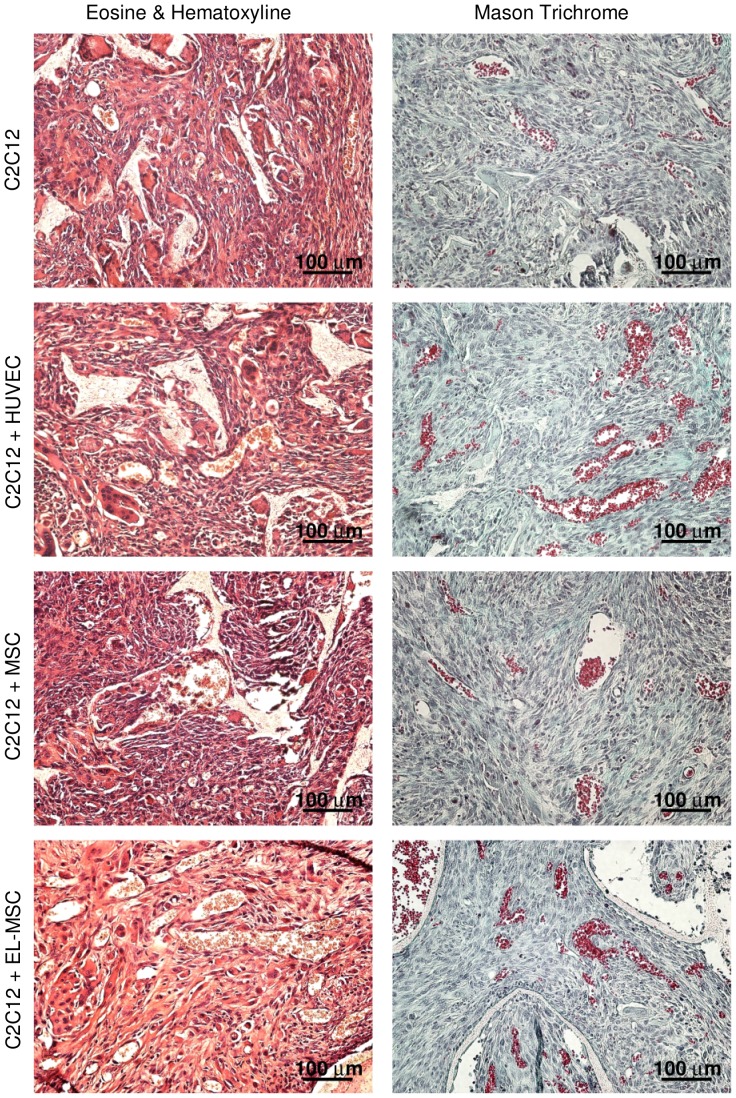
In vivo vascularization of polymeric construct combined with Matrigel and cells. Vessel-like network formation in vitro in 3D constructs. Tissue construct sections were stained with eosin (orange for tissue, red for erythrocytes) and counterstained with hematoxylin (brown) or with Masson’s trichrome staining (blue or green for collagen, red for erythrocytes) and counterstained with hematoxylin (brown).

As we described above, MSCs expanded in endothelial medium undergo significant changes in shape. We hypothesized that these changes might influence the results of the Matrigel assay. In order to confirm this, we performed a Matrigel assay with naïve MSCs and MSCs expanded in EGM-2 (pre-differentiated MSCs). Both naïve MSCs as well as pre-differentiated MSCs were seeded onto Matrigel in basic medium or in EGM-2. We observed cell behavior on Matrigel in a time course study (Fig. 5) where we found that naïve MSCs seeded in basic medium start to form capillaries on Matrigel at an earlier time point than pre-differentiated MSCs but those capillaries were very unstable and disrupted after 24 hours. A similar situation was observed when naïve MSCs were seeded on Matrigel in EGM-2. In contrast, pre-differentiated MSCs started to form capillaries with a 20 hours delay compared to naïve MSCs. However, those capillaries grew thicker and created a more complex network during the next 7 days. This showed that the expansion phase in EGM-2 is crucial for the stability of capillary structures.

**Figure 10:**
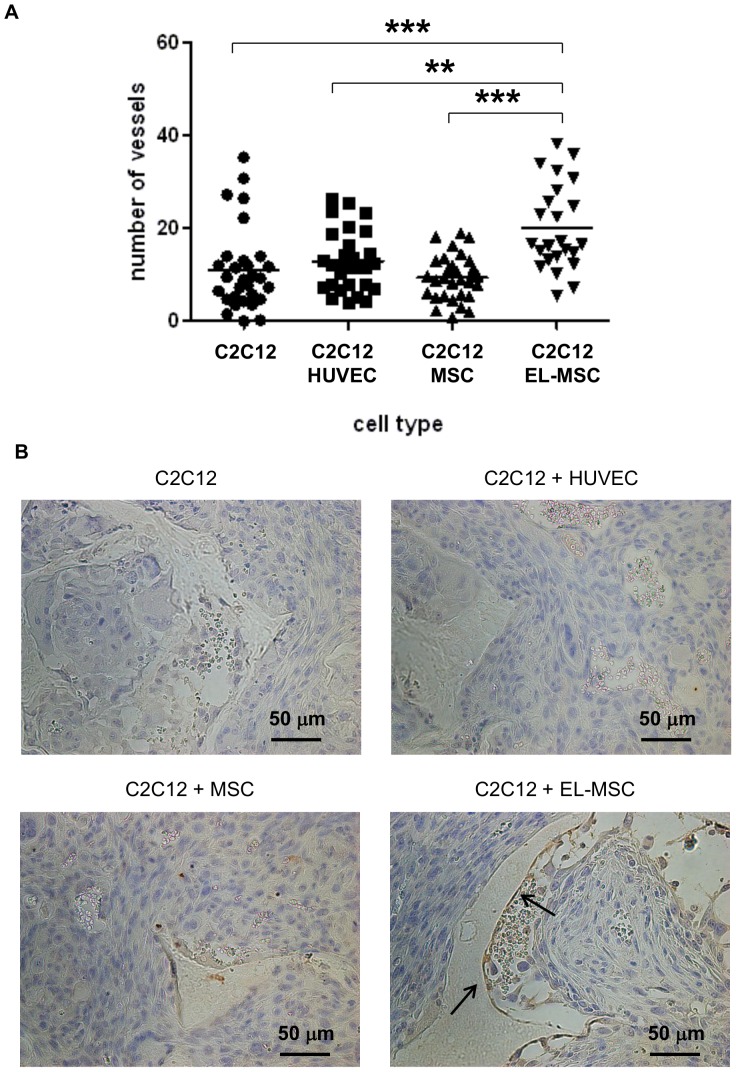
Quantitative analysis of vessels in polymeric constructs. Number of vessels per sample was quantified by four people blinded for the conditions (A). ** denotes statistical significance (P<0.01), *** denotes statistical significance (P<0.001). Tissue construct sections were stained with anti-human CD31 antibody (brown, indicated by black arrows) and counterstained with hematoxylin (blue) (B).

Quantification of the capillaries obtained in different conditions (Fig. 6) demonstrated a significant increase in the total tube length, total tube area, number of tubes and branching points when cells were expanded in EGM-2 even when the process of tube formation was performed in basic medium. This means that cells expanded in EGM-2 behave more like endothelial cells even when used in less promoting conditions (basic medium is not supplemented with growth factors as much as EGM-2). This is interesting when taking into account that endothelial cells need well supplemented medium to survive prolonged culture: HUVECs seeded in basic medium did not survive (data not shown).

### Endothelial Induction of MSCs

Since we did not observe endothelial marker expression in MSCs when growth factors and shear stress were applied we decided to test whether introduction of extracellular matrix could trigger an endothelial phenotype in MSCs. Cells cultured for 10 days in EGM-2 on a XYZ shaker were reseeded on Matrigel for another 24 hours. This short period of Matrigel culture allows for endothelial induction without the risk of MSCs modifying the gel itself [62]. Cells obtained in this way will be called endothelial-like MSCs (EL-MSCs).

### EL-MSCs Express Endothelial Markers

To characterize the phenotype of EL-MSCs, the expression of several endothelial genes, CD31, KDR and vWF, was assessed. hMSCs from 2 donors and iMSCs were used in this study. In all three cases the expression of CD31 and KDR was significantly higher in EL-MSCs than in naïve MSCs from the same source. CD31 expression was up-regulated between 15–60 times and KDR expression between 80–1000 times (Fig. 7A). In the case of vWF the expression was 5–6 times higher in hMSCs from both donors but not in case of the immortalized clone (Fig. 7A). To confirm the qPCR data, immunostaining was performed. iMSCs expanded in EGM-2, seeded on Matrigel for 24 h and recovered with dispase/trypsin solution were stained for CD31 and KDR. Staining directly on Matrigel was not possible due to the very high background signal generated by unspecific antibody binding to Matrigel. HUVECs and naïve iMSCs served as positive and negative control respectively. We observed cells positive for tested markers among EL-iMSCs whereas naïve iMSCs were negative (Fig. 7B). The specificity of the antibody for both markers was confirmed by HUVECs staining – cells recovered from Matrigel maintained round shape and did not spread on tissue culture plastic, which made the observation of characteristic staining on the border of neighboring cells very difficult.

The last characteristic of endothelial cells we tested was the ability of EL-MSCs to take up ac-LDL. iMSCs that were expanded in EGM-2 for 10 days and seeded on Matrigel for 24 hours were then recovered from the gel and reseeded on tissue culture plastic. Twenty-four hour later cells were incubated with ac-LDL for 4 hour, washed and imaged. Most of the cells were lost during this process. The cells that survived returned to a fibroblast-like morphology but were still able to take up ac-LDL. This uptake was however limited in the view of the number of positive cells when compared to HUVECs but was not observed at all in naïve MSCs (Fig. 7B).

### PLLA/PLGL Construct

We previously reported the induction of vessel networks in engineered tissue constructs using a three-dimensional coculture system consisting of cells seeded on porous and biodegradable polymer scaffolds [58]. The scaffolds were composed of 50% PLLA and 50% PLGL, with a pore size of 200±60 µm (Fig. 8A and 8B). We have previously vascularized muscle tissue with tissue engineered constructs combining PLLA/PLGL scaffolds, Matrigel and HUVECs [58]. To assess the angiogenic potential of EL-MSCs we have compared their ability to improve construct vascularization with that of HUVECs and naïve MSCs. C2C12 monoculture was used as negative control for angiogenic potential of constructs seeded with C2C12 with HUVECs, MSCs or EL-MSCs in 1∶1 ratio.

We showed that scaffolds filled with Matrigel without cells and cultured for 10 days in EGM-2 kept their structure (Fig. 8C). Hematoxylin/eosin staining performed on cross sections of constructs 10 days after cell seeding indicates that cells attached to and grew on the scaffolds in all four culture systems (Fig. 8D–G), no differences in cell density were detected. Elongated cells were observed in all used schemes, indicating cell-matrix interactions.

To observe the therapeutic potential of EL-MSCs we subcutaneously implanted tissue engineered constructs in immune-deficient mice (NMRI-nu, Harlan). The constructs were permeated with host blood vessels (Fig. 9) and the number of ingrown vessels were quantified. There were no significant differences between constructs seeded with C2C12 cells alone or in coculture with HUVECs or with naïve MSCs (Fig. 10A). Only in constructs seeded with C2C12 cells in coculture with EL-MSCs the number of vessels was significantly higher compared to all other tested conditions. These *in vivo* results showed that introduction of EL-MSCs in the implant improve construct vascularization and by that can promote cell survival in large grafts.

Staining of implants with anti-human specific endothelial antibody (anti-CD31) demonstrated that in the constructs seeded with EL-MSCs vessels lined with human cells were present (Fig. 10B). Moreover, these vessels contained intraluminal red blood cells suggesting that the vessels had anastomosed with the host vasculature.

## Discussion

Proper vascularization is essential for maintaining tissue well-being and functionality. It is also crucial for engineering of bone graft constructs, liver and many other tissues used for transplantation [63]–[65]. To provide extensive tissue or graft vascularization, a source of endothelial cells must be found. Those cells have to be available in large quantities and be able to create a vascular network within the tissue. Furthermore, this network should be both structurally and functionally appropriate.

Since BM-MSCs are widely used for bone tissue engineering, we decided to investigate whether we can use the same cell source for vascular network formation. In this study we used a number of techniques including immune-fluorescent imaging, qPCR and quantitative image analysis to examine endothelial differentiation of MSCs at the cellular and protein level. This study is the first to fully describe differentiation of MSCs into endothelial-like cells that perform in an *in vivo* study better than undifferentiated (naïve) MSCs.

BM-MSCs can be obtained in large quantities and further expanded *in vitro*. For most *in vivo* applications, a minimal number of cells, in the range of 10^8^–10^9^, are necessary [66] and this can easily be obtained from MSCs after only a few passages. We did not observe any serious loss of endothelial potential in cells expanded up to such a level. In the differentiation protocol MSCs were first cultured for 10 days in EGM-2 medium on an orbital shaker. The effect of this step was limited to changes in morphology and ability to perform in the Matrigel assay. After this culture period we did not observe a change either in marker expression or in the ability to take up ac-LDL. Nevertheless, this step was crucial for further performance in functional assays and differentiation. Functional properties of EGM-2-cultured MSCs were demonstrated using the Matrigel assay. MSCs that underwent 10 days of culture in EGM-2 medium performed with similar efficiency to endothelial cells (HUVECs) in Matrigel sprouting assay and much better than naïve MSCs. Interestingly, capillaries formed by MSCs were more stable than the ones formed by HUVECs – when applying prolongated culture, capillaries formed by HUVECs get disorganized whereas capillaries formed by MSCs remain stable. This can indicate that while forming capillaries MSCs can play both the role of endothelial cells that create vessels as well as the role of pericytes that stabilize those vessels [67]. The fact that MSCs can take the role of pericytes in various engineered constructs has already been shown by Chamberlain *et al* [68]. They reported that in co-culture with HUVECs on collagen modules MSCs became smooth muscle actin positive and migrated to surround the EC layer of the vessel (location typical for pericytes).

Pre-differentiated MSCs were also able to acquire endothelial characteristic (endothelial markers expression and ability to take up ac-LDL) after 24 h culture on Matrigel which was not the case when using naïve MSCs. This showed that growth supplements combined with shear force play an important role in triggering endothelial differentiation of MSCs, though this effect cannot be observed based on marker expression only. Although the molecular mechanisms of angiogenesis and vasculogenesis are currently not fully understood, there is evidence strongly supporting the crucial role of VEGF in both processes [69]. The expression of the main receptor for VEGF, KDR, did not increase in hMSCs after culture in EGM-2, but other studies showed that many key events in VEGF signaling occur inside endothelial cells and are regulated by endosomal receptor trafficking [70]. According to this model even basally expressed KDR can propagate the VEGF signaling. The VEGF-KDR complex is endocytosed directly after signal transduction and then proteolytic cleavage takes place, releasing the cytoplasmic KDR dimmer and making it available to form new signaling complexes. In our work we did not check for expression of Neuropilins and Ephiryns that play a crucial role in both VEGF and KDR endocytosis [71], [72] but we hypothesize that this can be the mechanism that triggers MSC differentiation towards endothelial lineages. This would be similar to the processes that occur in the embryo when the multipotent mesodermal progenitor cells differentiate *in situ* into endothelial cells during early stages of vascular development. VEGF, together with bFGF, is known to play a critical role in these events [73].

The input of Matrigel on pre-differentiated MSCs that leads to further differentiation towards endothelial cell phenotype can be explained based on the work of Lozito *et al.* [62]. They showed that change in the composition and crosslinking level of the matrix influences MSC differentiation towards endothelial and smooth muscle phenotype. Osawa *et al.* [74] showed that CD31 can be the mechanoresponsive molecule in case of endothelial cells, but we did not observe an increase in CD31 expression in MSCs before Matrigel stimulation. Therefore, two possible explanations of the effect of Matrigel on MSCs can be suggested. There can be another molecule on MSCs that acts as mechanoreceptor or there is a positive feedback loop between the ECM stimulation and CD31 expression in MSCs. Culture on Matrigel also induced KDR expression in MSCs which can further stimulate endothelial differentiation by increasing MSC sensitivity to VEGF present in culture medium. These results are similar to the ones obtained by Gu *et al*. [75] while studying murine embryonic stem cells. They showed that increased expression of endothelial markers is induced by extracellular matrix via CEACAM1, a glycoprotein involved in cell-cell adhesion. Therefore we can hypothesize that the role of Matrigel in our differentiation system is mainly to provide the environment in which MSCs can create cell-cell contact promoting endothelial differentiation. Nevertheless, those results suggest that careful examination of the MSC differentiation protocols is necessary as our knowledge concerning their signaling pathways is still limited.

CD31 staining performed on the constructs with EL-MSCs revealed the presence of human cells lining the walls of vessels. These vessels were fully functional as demonstrated by the presence of erythrocytes in the vessel lumens. This is a proof that EL-MSCs actively participated in building of these vessels. Nevertheless, there were also vessels present that were not human CD31 positive. This can suggest that the EL-MSC influence on vascularization is also due to their trophic effect on surrounding tissues. There is also the possibility that EL-MSCs create a network within the sample that is then gradually replaced by in-growing vessels. In that case the input of EL-MSCs can be limited to providing the route for host endothelial cells.

Although the primary purpose of this study was to differentiate hMSCs into endothelial-like cells, the results also serve as proof of concept for using bone marrow-derived hMSCs to create a vascularised graft. Our *in vivo* study showed that introduction of EL-MSCs in the engineered construct doubles the number of vessels in-growing in the construct 2 weeks after implantation. The fact that HUVECs did not induce vessel in-growth when compared to C2C12 or C2C12/MSC seeded constructs indicates that these endothelial cells are not able to create stable vascular structures without the support of other cells, pericytes or smooth muscle cells, which will stabilize newly formed blood vessels. Similar results were obtained by Levenberg *et al.* [58] where they have shown that the addition of embryonic fibroblasts promotes stable vessel formation. According to our results, EL-MSCs do not require the presence of an additional cell type to improve construct vascularization. Increased level of vascularization is crucial for the implantation of large grafts and the possibility to use the patient’s own cells to stimulate such an effect is a promising finding for a number of different applications. Further studies are required to verify whether differentiation of hMSCs prior to application can further improve performance of these cells in peripheral vascular disease (PVD) treatment as our data suggest.

This study also presents the opportunity of using iMSCs as model cells for studying the endothelial differentiation of MSC. This can be a useful tool for further study since it provides cells that are not affected by potential donor variation and isolation procedures.

In summary, hMSCs derived from bone marrow acquire several endothelial-like characteristics when cultured in endothelial cell growth supplement and exposed to shear force and extracellular matrix stimuli. These features include both phenotypical as well as functional characteristics. Furthermore, when applied *in vivo*, EL-MSCs show greater angiogenic potential than both naïve MSCs as well as truly endothelial cells, HUVECs, that are generally used as golden standard for vascularization studies. This study presents a facile protocol for MSC preconditioning that improves *in vivo* performance of these cells with respect to attracting neovascularization. We believe that this approach has potential applications in tissue engineering and provides a tool for various clinical studies where improved vascularization is desired.

To conclude, training MSCs *in vitro* can be an efficient way to manipulate the fate of these cells *in vivo*.

## References

[1] Friedenstein AJ, Chailakhyan RK, Latsinik NV, Panasyuk AF, Keiliss-Borok IV (1974) Stromal cells responsible for transferring the microenvironment of the hemopoietic tissues. Cloning in vitro and retransplantation in vivo. Transplantation 17: 331–340. 4150881 10.1097/00007890-197404000-00001

[2] Bellows CG, Aubin JE (1989) Determination of numbers of osteoprogenitors present in isolated fetal rat calvaria cells in vitro. Dev Biol 133: 8–13. 2707489 10.1016/0012-1606(89)90291-1

[3] Penfornis P, Pochampally R (2011) Isolation and expansion of mesenchymal stem cells/multipotential stromal cells from human bone marrow. Methods Mol Biol 698: 11–21. 21431507 10.1007/978-1-60761-999-4_2

[4] Cowan CM, Shi YY, Aalami OO, Chou YF, Mari C, et al (2004) Adipose-derived adult stromal cells heal critical-size mouse calvarial defects. Nat Biotechnol 22: 560–567. 15077117 10.1038/nbt958

[5] Oreffo RO, Bennett A, Carr AJ, Triffitt JT (1998) Patients with primary osteoarthritis show no change with ageing in the number of osteogenic precursors. Scand J Rheumatol 27: 415–424. 9855211 10.1080/030097498442235

[6] Miao Z, Jin J, Chen L, Zhu J, Huang W, et al (2006) Isolation of mesenchymal stem cells from human placenta: comparison with human bone marrow mesenchymal stem cells. Cell Biol Int 30: 681–687. 16870478 10.1016/j.cellbi.2006.03.009

[7] Chai YC, Roberts SJ, Desmet E, Kerckhofs G, van Gastel N, et al (2012) Mechanisms of ectopic bone formation by human osteoprogenitor cells on CaP biomaterial carriers. Biomaterials 33: 3127–3142. 22269651 10.1016/j.biomaterials.2012.01.015

[8] Friedenstein AJ, Chailakhjan RK, Lalykina KS (1970) The development of fibroblast colonies in monolayer cultures of guinea-pig bone marrow and spleen cells. Cell Tissue Kinet 3: 393–403. 5523063 10.1111/j.1365-2184.1970.tb00347.x

[9] Kanda Y, Hinata T, Kang SW, Watanabe Y (2011) Reactive oxygen species mediate adipocyte differentiation in mesenchymal stem cells. Life Sci 89: 250–258. 21722651 10.1016/j.lfs.2011.06.007

[10] Bruder SP, Jaiswal N, Haynesworth SE (1997) Growth kinetics, self-renewal, and the osteogenic potential of purified human mesenchymal stem cells during extensive subcultivation and following cryopreservation. J Cell Biochem 64: 278–294. 9027588 10.1002/(sici)1097-4644(199702)64:2<278::aid-jcb11>3.0.co;2-f

[11] Bruder SP, Kurth AA, Shea M, Hayes WC, Jaiswal N, et al (1998) Bone regeneration by implantation of purified, culture-expanded human mesenchymal stem cells. J Orthop Res 16: 155–162. 9621889 10.1002/jor.1100160202

[12] Siddappa R, Fernandes H, Liu J, van Blitterswijk C, de Boer J (2007) The response of human mesenchymal stem cells to osteogenic signals and its impact on bone tissue engineering. Curr Stem Cell Res Ther 2: 209–220. 18220904 10.2174/157488807781696267

[13] Pittenger MF, Mackay AM, Beck SC, Jaiswal RK, Douglas R, et al (1999) Multilineage potential of adult human mesenchymal stem cells. Science 284: 143–147. 10102814 10.1126/science.284.5411.143

[14] Dezawa M, Ishikawa H, Itokazu Y, Yoshihara T, Hoshino M, et al (2005) Bone marrow stromal cells generate muscle cells and repair muscle degeneration. Science 309: 314–317. 16002622 10.1126/science.1110364

[15] Kuroda Y, Kitada M, Wakao S, Nishikawa K, Tanimura Y, et al (2010) Unique multipotent cells in adult human mesenchymal cell populations. Proc Natl Acad Sci U S A 107: 8639–8643. 20421459 10.1073/pnas.0911647107 PMC2889306

[16] de la Garza-Rodea AS, van der Velde-van Dijke L, Boersma H, Goncalves MA, van Bekkum DW, et al.. (2011) Myogenic properties of human mesenchymal stem cells derived from three different sources. Cell Transplant. 10.3727/096368911X580554 21669036

[17] Faroni A, Mantovani C, Shawcross SG, Motta M, Terenghi G, et al (2011) Schwann-like adult stem cells derived from bone marrow and adipose tissue express gamma-aminobutyric acid type B receptors. J Neurosci Res 89: 1351–1362. 21618582 10.1002/jnr.22652

[18] Albersen M, Kendirci M, Van der Aa F, Hellstrom WJ, Lue TF, et al.. (2011) Multipotent Stromal Cell Therapy for Cavernous Nerve Injury-Induced Erectile Dysfunction. J Sex Med. 10.1111/j.1743-6109.2011.02556.x 22145667

[19] Steffenhagen C, Dechant FX, Oberbauer E, Furtner T, Weidner N, et al.. (2011) Mesenchymal Stem Cells Prime Proliferating Adult Neural Progenitors Toward an Oligodendrocyte Fate. Stem Cells Dev. 10.1089/scd.2011.0137 PMC3396148 22074360

[20] Keating A (2006) Mesenchymal stromal cells. Curr Opin Hematol 13: 419–425. 17053453 10.1097/01.moh.0000245697.54887.6f PMC3365862

[21] Chatterjea A, Meijer G, van Blitterswijk C, de Boer J (2010) Clinical application of human mesenchymal stromal cells for bone tissue engineering. Stem Cells Int 2010: 215625. 21113294 10.4061/2010/215625 PMC2989379

[22] Abdel-Latif A, Bolli R, Tleyjeh IM, Montori VM, Perin EC, et al (2007) Adult bone marrow-derived cells for cardiac repair: a systematic review and meta-analysis. Arch Intern Med 167: 989–997. 17533201 10.1001/archinte.167.10.989

[23] Wollert KC, Meyer GP, Lotz J, Ringes-Lichtenberg S, Lippolt P, et al (2004) Intracoronary autologous bone-marrow cell transfer after myocardial infarction: the BOOST randomised controlled clinical trial. Lancet 364: 141–148. 15246726 10.1016/S0140-6736(04)16626-9

[24] Poulsom R, Alison MR, Cook T, Jeffery R, Ryan E, et al (2003) Bone marrow stem cells contribute to healing of the kidney. J Am Soc Nephrol 14 Suppl 1S48–54. 12761239 10.1097/01.asn.0000068162.02174.29

[25] Alison MR, Poulsom R, Jeffery R, Dhillon AP, Quaglia A, et al (2000) Hepatocytes from non-hepatic adult stem cells. Nature 406: 257. 10917519 10.1038/35018642

[26] Aranguren XL, Verfaillie CM, Luttun A (2009) Emerging hurdles in stem cell therapy for peripheral vascular disease. J Mol Med (Berl) 87: 3–16. 18712330 10.1007/s00109-008-0394-3

[27] Franz RW, Parks A, Shah KJ, Hankins T, Hartman JF, et al (2009) Use of autologous bone marrow mononuclear cell implantation therapy as a limb salvage procedure in patients with severe peripheral arterial disease. J Vasc Surg 50: 1378–1390. 19837539 10.1016/j.jvs.2009.07.113

[28] Colton CK (1995) Implantable biohybrid artificial organs. Cell Transplant 4: 415–436. 7582573 10.1177/096368979500400413

[29] Zumstein A, Mathieu O, Howald H, Hoppeler H (1983) Morphometric analysis of the capillary supply in skeletal muscles of trained and untrained subjects–its limitations in muscle biopsies. Pflugers Arch 397: 277–283. 6889095 10.1007/BF00580261

[30] Radisic M, Deen W, Langer R, Vunjak-Novakovic G (2005) Mathematical model of oxygen distribution in engineered cardiac tissue with parallel channel array perfused with culture medium containing oxygen carriers. Am J Physiol Heart Circ Physiol 288: H1278–1289. 15539422 10.1152/ajpheart.00787.2004

[31] Hung HS, Shyu WC, Tsai CH, Hsu SH, Lin SZ (2009) Transplantation of endothelial progenitor cells as therapeutics for cardiovascular diseases. Cell Transplant 18: 1003–1012. 19650968 10.3727/096368909X12483162196683

[32] Asahara T, Murohara T, Sullivan A, Silver M, van der Zee R, et al (1997) Isolation of putative progenitor endothelial cells for angiogenesis. Science 275: 964–967. 9020076 10.1126/science.275.5302.964

[33] Ingram DA, Mead LE, Tanaka H, Meade V, Fenoglio A, et al (2004) Identification of a novel hierarchy of endothelial progenitor cells using human peripheral and umbilical cord blood. Blood 104: 2752–2760. 15226175 10.1182/blood-2004-04-1396

[34] Masuda H, Alev C, Akimaru H, Ito R, Shizuno T, et al (2011) Methodological development of a clonogenic assay to determine endothelial progenitor cell potential. Circ Res 109: 20–37. 21566217 10.1161/CIRCRESAHA.110.231837

[35] Melero-Martin JM, Khan ZA, Picard A, Wu X, Paruchuri S, et al (2007) In vivo vasculogenic potential of human blood-derived endothelial progenitor cells. Blood 109: 4761–4768. 17327403 10.1182/blood-2006-12-062471

[36] Schatteman GC, Awad O (2003) In vivo and in vitro properties of CD34+ and CD14+ endothelial cell precursors. Adv Exp Med Biol 522: 9–16. 12674206 10.1007/978-1-4615-0169-5_2

[37] Schatteman GC, Dunnwald M, Jiao C (2007) Biology of bone marrow-derived endothelial cell precursors. Am J Physiol Heart Circ Physiol 292: H1–18. 16980351 10.1152/ajpheart.00662.2006

[38] Ensley AE, Nerem RM, Anderson DE, Hanson SR, Hinds MT (2011) Fluid Shear Stress Alters the Coagulation Potential of Endothelial Outgrowth Cells. Tissue Eng Part A. 10.1089/ten.tea.2010.0290 PMC3246409 21787250

[39] Rouwkema J, de Boer J, Van Blitterswijk CA (2006) Endothelial cells assemble into a 3-dimensional prevascular network in a bone tissue engineering construct. Tissue Eng 12: 2685–2693. 16995802 10.1089/ten.2006.12.2685

[40] Szoke K, Beckstrom KJ, Brinchmann JE (2011) Human adipose tissue as a source of cells with angiogenic potential. Cell Transplant. 10.3727/096368911X580518 21669039

[41] Bianco P, Gehron Robey P (2000) Marrow stromal stem cells. J Clin Invest 105: 1663–1668. 10862779 10.1172/JCI10413 PMC378520

[42] Bala K, Ambwani K, Gohil NK (2011) Effect of different mitogens and serum concentration on HUVEC morphology and characteristics: Implication on use of higher passage cells. Tissue Cell 43: 216–222. 21511321 10.1016/j.tice.2011.03.004

[43] Terramani TT, Eton D, Bui PA, Wang Y, Weaver FA, et al (2000) Human macrovascular endothelial cells: optimization of culture conditions. In Vitro Cell Dev Biol Anim 36: 125–132. 10718369 10.1290/1071-2690(2000)036<0125:HMECOO>2.0.CO;2

[44] Yang N, Li D, Jiao P, Chen B, Yao S, et al (2011) The characteristics of endothelial progenitor cells derived from mononuclear cells of rat bone marrow in different culture conditions. Cytotechnology 63: 217–226. 21331655 10.1007/s10616-010-9329-2 PMC3081048

[45] Davies PF, Mundel T, Barbee KA (1995) A mechanism for heterogeneous endothelial responses to flow in vivo and in vitro. J Biomech 28: 1553–1560. 8666594 10.1016/0021-9290(95)00102-6

[46] Zhang P, Baxter J, Vinod K, Tulenko TN, Di Muzio PJ (2009) Endothelial differentiation of amniotic fluid-derived stem cells: synergism of biochemical and shear force stimuli. Stem Cells Dev 18: 1299–1308. 19508152 10.1089/scd.2008.0331 PMC3139996

[47] Wang H, Riha GM, Yan S, Li M, Chai H, et al (2005) Shear stress induces endothelial differentiation from a murine embryonic mesenchymal progenitor cell line. Arterioscler Thromb Vasc Biol 25: 1817–1823. 15994439 10.1161/01.ATV.0000175840.90510.a8

[48] Yamamoto K, Sokabe T, Watabe T, Miyazono K, Yamashita JK, et al (2005) Fluid shear stress induces differentiation of Flk-1-positive embryonic stem cells into vascular endothelial cells in vitro. Am J Physiol Heart Circ Physiol 288: H1915–1924. 15576436 10.1152/ajpheart.00956.2004

[49] Bai K, Huang Y, Jia X, Fan Y, Wang W (2010) Endothelium oriented differentiation of bone marrow mesenchymal stem cells under chemical and mechanical stimulations. J Biomech 43: 1176–1181. 20022602 10.1016/j.jbiomech.2009.11.030

[50] Kniazeva E, Kachgal S, Putnam AJ (2011) Effects of extracellular matrix density and mesenchymal stem cells on neovascularization in vivo. Tissue Eng Part A 17: 905–914. 20979533 10.1089/ten.tea.2010.0275 PMC3063702

[51] Cao Y, Sun Z, Liao L, Meng Y, Han Q, et al (2005) Human adipose tissue-derived stem cells differentiate into endothelial cells in vitro and improve postnatal neovascularization in vivo. Biochem Biophys Res Commun 332: 370–379. 15896706 10.1016/j.bbrc.2005.04.135

[52] Fischer LJ, McIlhenny S, Tulenko T, Golesorkhi N, Zhang P, et al (2009) Endothelial differentiation of adipose-derived stem cells: effects of endothelial cell growth supplement and shear force. J Surg Res 152: 157–166. 19883577 10.1016/j.jss.2008.06.029 PMC2773556

[53] Oswald J, Boxberger S, Jorgensen B, Feldmann S, Ehninger G, et al (2004) Mesenchymal stem cells can be differentiated into endothelial cells in vitro. Stem Cells 22: 377–384. 15153614 10.1634/stemcells.22-3-377

[54] Silva GV, Litovsky S, Assad JA, Sousa AL, Martin BJ, et al (2005) Mesenchymal stem cells differentiate into an endothelial phenotype, enhance vascular density, and improve heart function in a canine chronic ischemia model. Circulation 111: 150–156. 15642764 10.1161/01.CIR.0000151812.86142.45

[55] Liu JW, Dunoyer-Geindre S, Serre-Beinier V, Mai G, Lambert JF, et al (2007) Characterization of endothelial-like cells derived from human mesenchymal stem cells. J Thromb Haemost 5: 826–834. 17229052 10.1111/j.1538-7836.2007.02381.x

[56] Doorn J, Siddappa R, van Blitterswijk C, De Boer J (2011) Forskolin enhances in vivo bone formation by human mesenchymal stromal cells. Tissue Eng Part A. 10.1089/ten.TEA.2011.0312 21942968

[57] Both SK, van der Muijsenberg AJ, van Blitterswijk CA, de Boer J, de Bruijn JD (2007) A rapid and efficient method for expansion of human mesenchymal stem cells. Tissue Eng 13: 3–9. 17518576 10.1089/ten.2005.0513

[58] Levenberg S, Rouwkema J, Macdonald M, Garfein ES, Kohane DS, et al (2005) Engineering vascularized skeletal muscle tissue. Nat Biotechnol 23: 879–884. 15965465 10.1038/nbt1109

[59] Ning H, Liu G, Lin G, Yang R, Lue TF, et al (2009) Fibroblast growth factor 2 promotes endothelial differentiation of adipose tissue-derived stem cells. J Sex Med 6: 967–979. 19207272 10.1111/j.1743-6109.2008.01172.x PMC2893032

[60] Rodriguez LG, Wu X, Guan JL (2005) Wound-healing assay. Methods Mol Biol 294: 23–29. 15576902 10.1385/1-59259-860-9:023

[61] Carmeliet P (2000) VEGF gene therapy: stimulating angiogenesis or angioma-genesis? Nat Med 6: 1102–1103. 11017137 10.1038/80430

[62] Lozito TP, Taboas JM, Kuo CK, Tuan RS (2009) Mesenchymal stem cell modification of endothelial matrix regulates their vascular differentiation. J Cell Biochem 107: 706–713. 19415686 10.1002/jcb.22166

[63] Hoganson DM, Pryor HI 2nd, Vacanti JP (2008) Tissue engineering and organ structure: a vascularized approach to liver and lung. Pediatr Res 63: 520–526. 18427297 10.1203/01.pdr.0000305879.38476.0c

[64] Nomi M, Atala A, Coppi PD, Soker S (2002) Principals of neovascularization for tissue engineering. Mol Aspects Med 23: 463–483. 12385748 10.1016/s0098-2997(02)00008-0

[65] Rafii S, Lyden D (2003) Therapeutic stem and progenitor cell transplantation for organ vascularization and regeneration. Nat Med 9: 702–712. 12778169 10.1038/nm0603-702

[66] McGuigan AP, Bruzewicz DA, Glavan A, Butte MJ, Whitesides GM (2008) Cell encapsulation in sub-mm sized gel modules using replica molding. PLoS One 3: e2258. 18493609 10.1371/journal.pone.0002258 PMC2376064

[67] da Silva Meirelles L, Caplan AI, Nardi NB (2008) In search of the in vivo identity of mesenchymal stem cells. Stem Cells 26: 2287–2299. 18566331 10.1634/stemcells.2007-1122

[68] Chamberlain MD, Gupta R, Sefton MV (2012) Bone marrow-derived mesenchymal stromal cells enhance chimeric vessel development driven by endothelial cell-coated microtissues. Tissue Eng Part A 18: 285–294. 21861779 10.1089/ten.tea.2011.0393 PMC3267971

[69] Carmeliet P, Ferreira V, Breier G, Pollefeyt S, Kieckens L, et al (1996) Abnormal blood vessel development and lethality in embryos lacking a single VEGF allele. Nature 380: 435–439. 8602241 10.1038/380435a0

[70] Eichmann A, Simons M (2012) VEGF signaling inside vascular endothelial cells and beyond. Curr Opin Cell Biol. 10.1016/j.ceb.2012.02.002 PMC4030755 22366328

[71] Ballmer-Hofer K, Andersson AE, Ratcliffe LE, Berger P (2011) Neuropilin-1 promotes VEGFR-2 trafficking through Rab11 vesicles thereby specifying signal output. Blood 118: 816–826. 21586748 10.1182/blood-2011-01-328773

[72] Erskine L, Reijntjes S, Pratt T, Denti L, Schwarz Q, et al (2011) VEGF signaling through neuropilin 1 guides commissural axon crossing at the optic chiasm. Neuron 70: 951–965. 21658587 10.1016/j.neuron.2011.02.052 PMC3114076

[73] Cross MJ, Claesson-Welsh L (2001) FGF and VEGF function in angiogenesis: signalling pathways, biological responses and therapeutic inhibition. Trends Pharmacol Sci 22: 201–207. 11282421 10.1016/s0165-6147(00)01676-x

[74] Osawa M, Masuda M, Kusano K, Fujiwara K (2002) Evidence for a role of platelet endothelial cell adhesion molecule-1 in endothelial cell mechanosignal transduction: is it a mechanoresponsive molecule? J Cell Biol 158: 773–785. 12177047 10.1083/jcb.200205049 PMC2174013

[75] Gu A, Tsark W, Holmes KV, Shively JE (2009) Role of Ceacam1 in VEGF induced vasculogenesis of murine embryonic stem cell-derived embryoid bodies in 3D culture. Exp Cell Res 315: 1668–1682. 19285068 10.1016/j.yexcr.2009.02.026 PMC2745895

